# Collaborative care for patients with depression and diabetes mellitus: a systematic review and meta-analysis

**DOI:** 10.1186/1471-244X-13-260

**Published:** 2013-10-14

**Authors:** Yafang Huang, Xiaoming Wei, Tao Wu, Rui Chen, Aimin Guo

**Affiliations:** 1 School of General Practice and Continuing Education, Capital Medical University, Beijing 100069, China; 2 Datun Community Health Service Center, Beijing Anzhen Hospital, Capital Medical University, Beijing 100029, China; 3 Research Department, Beijing Anzhen Hospital, Capital Medical University, Beijing 100029, China

**Keywords:** Collaborative care, Depression, Diabetes mellitus, Systematic review, Meta-analysis

## Abstract

**Background:**

Diabetic patients with depression are often inadequately treated within primary care. These comorbid conditions are associated with poor outcomes. The aim of this systematic review was to examine whether collaborative care can improve depression and diabetes outcomes in patients with both depression and diabetes.

**Methods:**

Medline, Embase, Cochrane library and PsyINFO were systematically searched to identify relevant publications. All randomized controlled trials of collaborative care for diabetic patients with depression of all ages who were reported by depression treatment response, depression remission, hemoglobin A1c (HbA1c) values, adherence to antidepressant medication and/or oral hypoglycemic agent were included. Two authors independently screened search results and extracted data from eligible studies. Dichotomous and continuous measures of outcomes were combined using risk ratios (RRs) and mean differences (MDs) with 95% confidence intervals (CIs) either by fixed or random-effects models.

**Results:**

Eight studies containing 2,238 patients met the inclusion criteria. Collaborative care showed a significant improvement in depression treatment response (RR = 1.33, 95% CI = 1.05-1.68), depression remission (adjusted RR = 1.53, 95% CI =1.11-2.12), higher rates of adherence to antidepressant medication (RR = 1.79, 95% CI = 1.19-2.69) and oral hypoglycemic agent (RR = 2.18, 95% CI = 1.61-2.96), but indicated a non-significant reduction in HbA1c values (MD = -0.13, 95% CI = -0.46-0.19).

**Conclusions:**

Improving depression care in diabetic patients is very necessary and important. Comparing with usual care, collaborative care was associated with significantly better depressive outcomes and adherence in patients with depression and diabetes. These findings emphasize the implications for collaborative care of diabetic patients with depression in the future.

## Background

Depression and diabetes mellitus (DM) are two of the most prevalent chronic diseases around the world, which frequently co-occur [1-5]. Approximately 20% of patients with DM meet diagnostic criteria for depression [4,5]. Diabetic patients with depression are associated with decreased glycated hemoglobin control, lower adherence to diet, exercise and taking medications, comparing with those without depression [6-9]. Moreover, depression had an increased risk in diabetes development and adverse diabetes outcomes, such like microvascular and macrovascular complications [10,11].

Patients with diabetes and depression are usually poorly managed in primary care [7,12]. Depression is associated with failures to detect and diagnose in diabetic patients [13]. Diabetes also weaken the effectiveness of depression treatments [14,15]. Depression care needs to be improved especially in people with diabetes and vice versa. The most common method of treatments for diabetes and depression in primary care are taking medications of oral hypoglycemic agents and antidepressants. However, diabetes patients with depression are more likely to have problems and concerns with medication, such like fear of side effects and addiction, than those patients without depression [1,15-17]. Conventional psychological interventions fail to improve both physical and mental health outcomes in diabetes patients with depression [18].

Collaborative care is a new model pointing out coordinated care management in primary practices, which involving primary care physicians, nurses and other specialists or professionals who provided patient-orientated and guideline-based management to patients at the primary care level [12,19-22]. It is originally conducted on depressive patients. More studies of collaborative care have been diversified to those patients with chronic illnesses [19,23]. Nowadays, collaborative care attracts a worldwide interest in its potential effectiveness in achieving certain clinically improvements and public health benefits [12,19-21,24,25]. Several randomized controlled trials (RCTs) indicated that collaborative care significantly improved control of both depression and diabetes [19,26,27]. However, some studies concluded that collaborative care improved depression outcome alone [23,28]. There is no consensus on these results. We still do not know whether collaborative care work as a truly integrated intervention that improve both depression and diabetes outcomes.

We therefore conducted a systematic review and meta-analysis to examine whether a primary care based collaborative care would improve depression and diabetes outcomes in patients with both depression and diabetes.

## Methods

### Publication search

Literature searches were conducted through March 27, 2013 using the electronic databases Medline (1946 to present), Embase (1980 to present), Cocharne library (present) and PsycINFO (1806 to present). The detailed search strategies were shown in Additional file 1. No restriction was placed on type of language. We also screened the references from retrieved articles and reviews to identify additional articles which met the eligibility criteria.

### Study selection

Studies that met the following criteria were included in this meta-analysis: (1) Participants: both male and female patients of any age, with a diagnosis of both depression and diabetes. Diagnosis of depression was according to one of the following: a, diagnosis made by primary care physicians; b, current prescription for an antidepressant; c, diagnosis according to International Classification of Diseases, Ninth Revision (ICD-9) code, Diagnostic and Statistical Manual (DSM) and/or Research Diagnostic Criteria (RDC); d, assessment through clinician-rated and/or self-rated validated instruments, for example, patient health questionnaire (PHQ); e, diagnosis made through structured psychiatric interview. Diagnosis of diabetes was according to one of the following: a, diagnosis made by primary care physicians; b current prescription for a glucose lowering medication; c, diagnosis according to ICD-9 code; d, diagnosis according to laboratory result. (2) Type of intervention: although there was considerable variability in the exact nature of the intervention [29], we regarded that if it fulfilled the following four criteria as the intervention of collaborative care: a, a multi-professional patient care; b, a structured management plan; c, scheduled patient follow up; d, enhanced inter-professional communication [30]. (3) Type of control: we regarded it as control according to one of the following: a. no additional intervention was provided; b. usual care was provided in the control group; c. enhanced usual care was provided in the control group. (4) Type of outcome measurements: the studies were included if one of the following outcomes were reported in the original article: a, depression treatment response; b, depression remission; c, hemoglobin A1c (HbA1c) control; d, adherence to medication (including adherence of oral hypoglycemic agents and/or antidepressants); (5) Type of studies: only clinical randomized controlled trials (RCTs) or cluster RCTs were eligible; (6) Setting: primary care settings.

**Figure 1:**
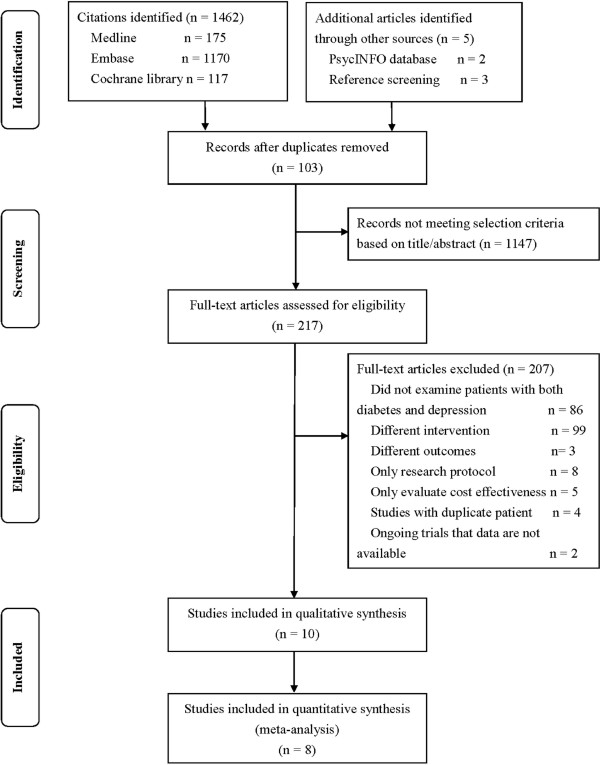
Flow chart of study selection.

Studies that did not meet the above criteria were excluded. In addition, those duplicated publications were excluded. Two authors (YH TW) independently evaluated the articles for inclusion. Any discrepancies were resolved by further discussion and consultation of a third author (XW). The selection process by means of a flow chart was presented in Figure 1.

### Data extraction

A standardized data extraction form was used. The following information was extracted: first author name, publication year, country of corresponding author, study design, study period, age, gender, ethnicity, sample size (intervention/control), length of follow-up, conflicts of interest (Table 1), inclusion criteria of patients, description of “collaborative care” and “control”, main outcomes (see Additional file 2). All the corresponding authors of studies included in qualitative synthesis were contacted by e-mail a maximum of 3 times to obtain additional information. However, no information was received from the original authors.

**Table 1:** Characteristic of the included studies

**Author**	**Year**	**Country**	**Study design**	**Study period conducted**	**Setting**	**Age, mean (y)**	**Gender, male (%)**	**Ethnicity, white (%)**	**Randomized sample size (Intervention/control)**	**Follow-up**	**Loss to follow-up based on primary outcome (%)**	**Intention-to-treat analysis**
Bogner et al. [26]	2012	US	RCT	From April 2010 to April 2011	3 Primary care practices	57	58 (32)	65 (36)	92/88	6, 12 wk	Intervention group: 0/92 (0%)	Used
											Control group: 0/88 (0%)	
Bogner et al. [27]	2010	US	RCT	From April 2007 to June 2008	1 Primary care practice	60	9 (16)	0 (0)^*^	29/29	6 wk	Intervention group: 0/29 (0%)	Not used
											Control group: 0/29 (0%)	
Ciechanowski et al. [31]	2006	US	RCT	Not clear	9 health maintenance organization clinics	58	112 (36)	248 (78)	160/164	3, 6, 12 mo	Intervention group: 0/160 (0%)	Used
											Control group: 0/164 (0%)	
Ell et al. [32]	2011	US	RCT	From August 2005 to August 2007	2 public safety-net clinics	Not clear^**^	69 (18)	Not clear^***^	193/194	6, 12, 18, 24 mo	Intervention group: 55/193 (28.5%)	Used
											Control group: 68/194 (35.1%)	
Katon et al. [19]	2010	US	RCT	From May 2007 to October 2009	14 Primary care clinics	57	103 (48)	169 (79)	106/108	6, 12 mo	Intervention group: 12/106 (11.3%)	Used
											Control group: 16/108 (14.8%)	
Katon et al. [23]	2004	US	RCT	From March 2001 to May 2002	9 Primary care clinics	58	115 (35)	260 (79)	164/165	3, 6, 9, 12 mo	Intervention group: 21/164 (13%)	Used
											Control group: 16/165 (9.7%)	
Kinder et al. [33]	2006	US	RCT	Not clear	Primary care clinics	58	115 (35)	248 (75)	164/165	6, 12 mo	Intervention group: 18/164 (11.0%)	Not used
											Control group: 23/165 (13.9%)	
Williams Jr et al. [28]	2004	US	RCT	From July 1999 to August 2001	18 Primary care clinics	70	194 (47)	265 (64)	205/212	3, 6, 12 mo	Intervention group: 5/205 (2.4%)	Used
											Control group: 19/212 (9.0%)	

Abbreviations: *mo* month, *SCL-20* Symptom Checklist-20, *RCT* Randomized Controlled Trial, *US* United States, *wk* week.

*100% African American.

**72% > =50 years.

***96% Hispanic.

### Quality assessment

The risk of bias for the included studies was assessed using the *Cochrane Collaboration’s tool for assessing risk of bias*[34]. Two authors (XW TW) independently assessed the methodological quality of RCTs using the Cochrane risk of bias tools. Any discrepancies were resolved by a third author (RC).

### Statistical analysis

Primary clinical outcomes of interest, evaluated at the end of follow up, 6 months and 12 months of follow up (during which patients were on collaborative care intervention) respectively, included depression treatment response (defined as 50% or more decrease in the Hopkins Symptoms Checklist-20 (SCL-20) score from base line); depression remission (defined as SCL-20 score less than 0.5); diabetes clinical outcomes (HbA1c values) (defined as HbA1c measures exposure of red blood cells to glucose during a 90-day period). Secondary outcomes were adherence (defined as the percentage of prescribed doses taken, calculated as the number of doses taken divided by the number of doses prescribed over the observation period * 100%) to antidepressant medication and oral hypoglycemic agent (which was dichotomized at a threshold of 80%) [35].

We used Revman software (version 5.2), which was available through the Cochrane Collaboration (http://www.cochrane.org). Heterogeneity was quantified with a Chi-square heterogeneity statistic and by means of I square, with a predefined significance threshold of 0.1 [36]. If a significant trend for heterogeneity was observed, a random effect model via generic inverse variance weighting was used to combine the effect [37]. Otherwise, we used a fixed-effects model to calculate the pooled effects. Results were expressed as the relative risks (RRs) with 95% confidence intervals (CIs) for dichotomous variables and mean differences (MDs) with 95% CI for continuous variables. Results were considered statistically significant when *P* < 0.05. The possibility of publication bias was initially planned to be evaluated by funnel plots, but not adopted eventually either because of the limited number of trials included or the significant heterogeneity among trials [38]. No additional analysis was performed. No protocol of the present review has been published or registered.

## Results

### Literature search

We identified 1,467 citations (Figure 1). After excluding 103 duplicate records, two authors (YH XW) screened 1,364 titles and abstracts to identify the potentially relevant studies. Totally 217 full-text articles were assessed for eligibility. Of these, 10 studies were included in qualitative synthesis [2,19,23,24,26,27,31-33]. A total of 8 articles met the final eligibility criteria for meta-analysis [19,23,26,27,31-33]. The detailed selection process was described in Figure 1.

### Characteristics of the included studies

Table 1 summarized the basic characteristics of the included randomized controlled trials. There were 8 studies with a total of 2,238 patients with both depression and diabetes, which compared collaborative care with usual care [19,23,26,27,31-33]. All trials were from the United States. One trial included only African Americans [27]. Two trials included only old patients (aged or above 50 and 60 respectively) [27,28]. Three trials used enhanced usual care for the control group [19,31,32] and five trials used normal usual care [23,26-28,33]. Duration of trials varied from 13 months to 30 months.

Random sequence generation [23,28,31,32] and allocation concealment [19,23,28,32] were described adequately in four studies respectively. Blinding of patients was not possible. Therefore, all studies had high risk of bias in blinding. Five studies [19,23,28,36,38,] described adequate blinding of outcome assessment. Two studies [19,23] did not provide sufficient information for assessment of incomplete outcome data. Six studies [19,23,28,31-33] reported all expected outcomes. The detailed risk of bias of all included trials was shown in Table 2.

**Table 2:** The risk of bias of included studies

**Studies**	**Year**	**Selection bias**		**Performance bias**	**Detection bias**	**Attrition bias**	**Reporting bias**	**Other bias**
		**Random sequence generation**	**Allocation concealment**	**Blinding of participants and personnel**	**Blinding of outcome assessment**	**Incomplete outcome data**	**Selective reporting**	**Anything else, ideally prespecified**
Bogner et al. [26]	2012	U	U	H	U	L	U	U
Bogner et al. [27]	2010	U	U	H	U	L	U	U
Ciechanowski et al. [31]	2006	L	U	H	L	L	L	U
Ell et al. [32]	2011	L	L	H	L	L	L	U
Katon et al. [19]	2010	U	L	H	L	U	L	U
Katon et al. [23]	2004	L	L	H	L	U	L	U
Kinder et al. [33]	2006	U	U	H	U	L	L	U
Williams Jr et al. [28]	2004	L	L	H	L	L	L	U

*H* high risk of bias, *L* low risk of bias, *U* unclear risk of bias.

### Depression treatment response and depression remission at the end of follow up

**Figure 2:**
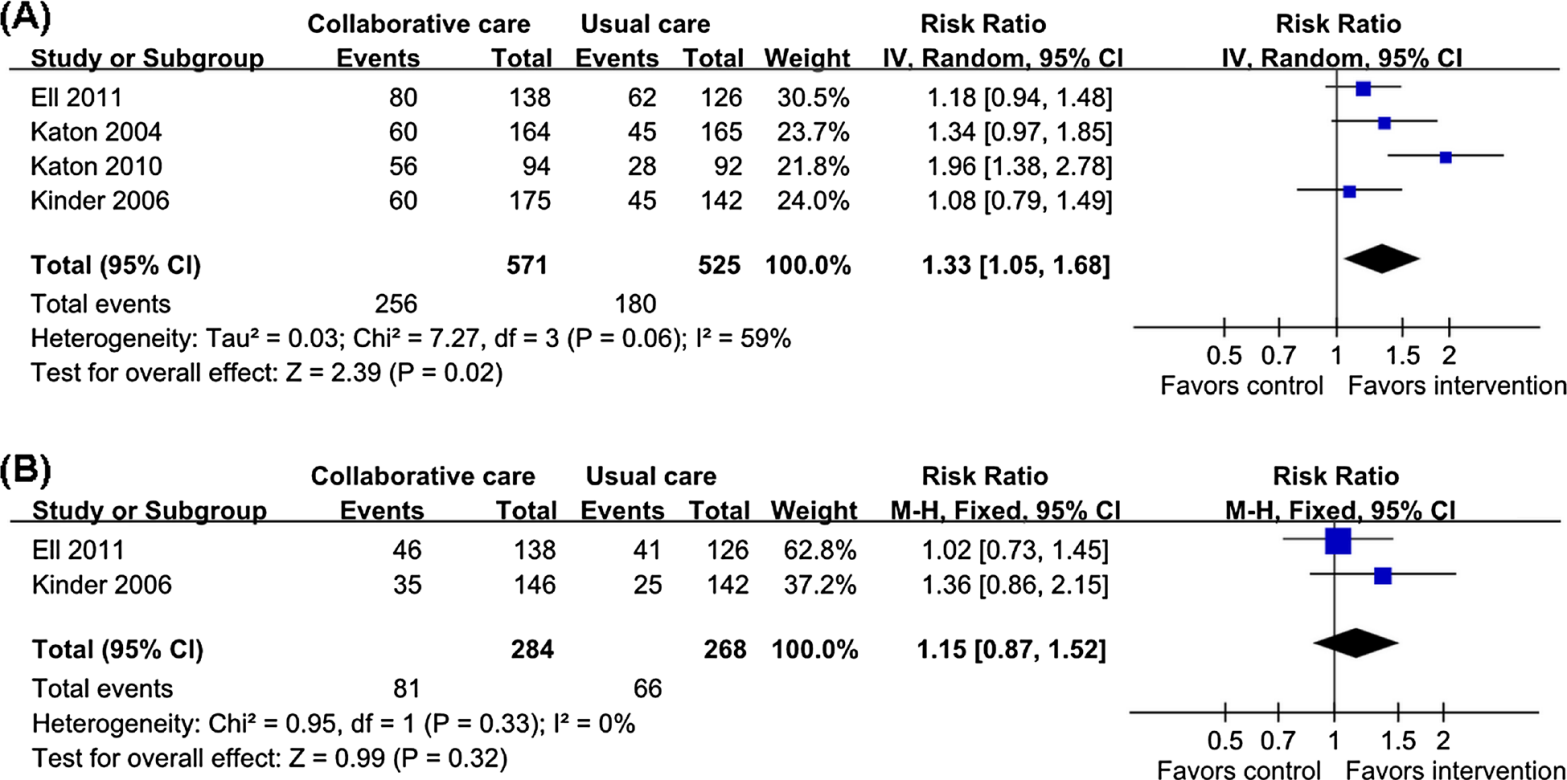
**Depression treatment response and remission at the end of follow up.** Depression treatment response and remission estimated from meta-analysis of diabetic depression patients with collaborative care (intervention group) versus usual care (control group). **(A)** Number of patients with treatment response. **(B)** Number of patients with depression remission.

Four trials provided information on treatment response rate to calculate the overall effect size, with 1,096 patients [19,23,32,33]. The length treatment ranged from 12 to 24 month. All of these trials reported an increased treatment response rate in the collaborative group, and one was significant (RR = 1.96, 95% CI = 1.38-2.78) [19]. Pooling of the mean proportion showed that 44.8% of patients in intervention group and 34.3% of patients in control group had treatment responses. The meta-analysis showed that collaborative care was associated with a significant increase in treatment response rate at the end of follow up (RR = 1.33, 95% CI = 1.05-1.68; *P =* 0.06 for heterogeneity; I^2^ = 59%) (Figure 2A). The RR of 1.33 indicated a 33% relative increase in treatment response rate was added to collaborative care. There was significant heterogeneity in the studies.

Two trials reported information on depression remission rate [32,33]. Of these, one showed a significant improvement on remission rate in collaborative care group at the end of the 24 month’s treatment (adjusted RR = 1.53, 95% CI =1.11-2.12) [32]. Another reported a non-significant improvement [33]. There were 552 patients to calculate the overall effect size. The meta-analysis indicated that there was a non-significant effect of collaborative care (RR = 1.15, 95% CI = 0.87-1.52; *P =* 0.33 for heterogeneity; I^2^ = 0%) (Figure 2B).

### Depression treatment response at 6 and 12 months follow up

**Figure 3:**
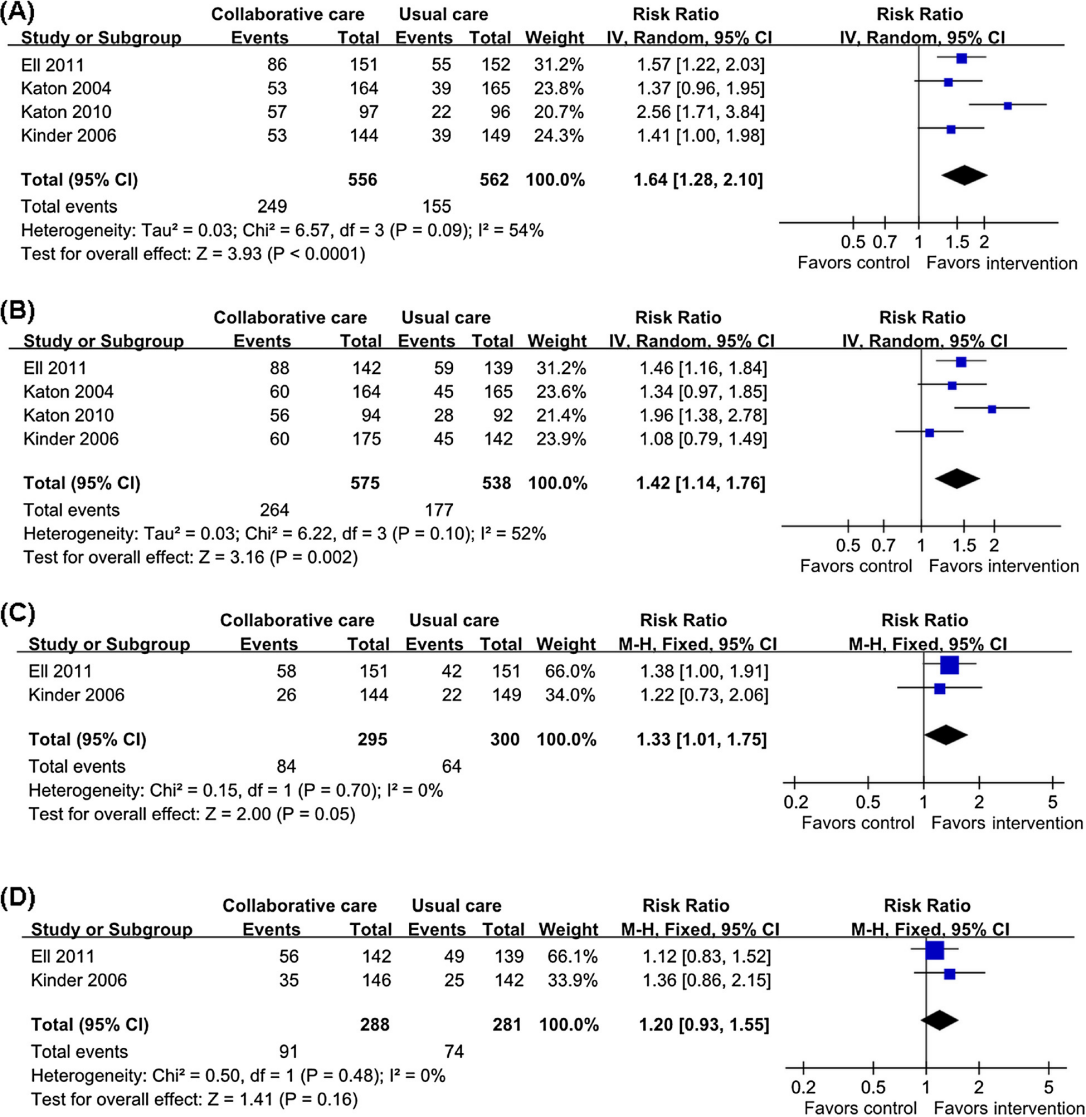
**Depression treatment response and remission at 6 and 12 months.** Depression treatment response and remission estimated from meta-analysis of diabetic depression patients with collaborative care (intervention group) versus usual care (control group). **(A)** Number of patients with treatment response at 6 months. **(B)** Number of patients with treatment response at 12 months. **(C)** Number of patients with depression remission at 6 months. **(D)** Number of patients with depression remission at 12 months.

Four trials evaluated treatment response rate at 6 months follow up, with totally 1,118 patients [19,23,32,33]. All reported an increased treatment response rate in the collaborative group, and three were significant. Combining the four trials also demonstrated a statistically significantly beneficial effect of collaborative care at 6 months follow up (RR = 1.64, 95% CI = 1.28-2.10; *P =* 0.09 for heterogeneity; I^2^ = 54%) (Figure 3A).

Four trials provided information to calculate the overall effect size of treatment response rate at 12 months follow up, with 1,344 patients [19,23,32,33]. All of these trials reported an increased treatment response rate in the collaborative group, and two were significant. The meta-analysis showed that collaborative care was associated with a significant increase in treatment response at 12 months follow up (RR = 1.42, 95% CI = 1.14-1.76; *P =* 0.10 for heterogeneity; I^2^ = 52%) (Figure 3B).

### Depression remission at 6 and 12 months follow up

Two trials reported depression remission rate at 6 months follow up, with an evaluation of 595 patients to calculate the overall effect size [32,33]. Both of the trials reported an increased treatment response rate in the collaborative group, neither of them was significant. However, the combined results indicated a significant increase with collaborative care (RR = 1.33, 95% CI = 1.01-1.75; *P =* 0.70 for heterogeneity; I^2^ = 0%) (Figure 3C).

Two trials reported information on depression remission at 12 months follow up, with an evaluation of 569 patients to calculate the overall effect size [32,33]. The meta-analysis resulted in a non-significant effect on depression remission at 12 months follow up (RR = 1.20, 95% CI = 0.93-1.55; *P =* 0.48 for heterogeneity; I^2^ = 0%) (Figure 3D).

### Diabetes clinical outcomes (HbA1c values) at the end of follow up

**Figure 4:**
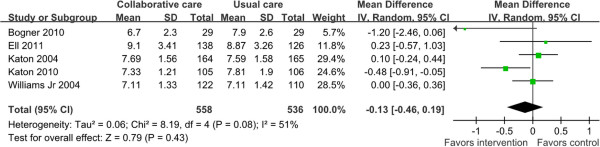
**Diabetes clinical outcomes (HbA1c values).** HbA1c values were estimated from meta-analysis of diabetic depression patients with collaborative care (intervention group) versus usual care (control group).

Seven trials reported HbA1c values. One trial [26] showed a significant reduction on HbA1c values in collaborative care group at the end of follow up. Another trial [24] showed that the percentage of patients with HbA1c values less than 7 was 60.9% and 35.7% in collaborative care group and usual care group respectively (no statistical analysis provided). The other five trials [19,23,27,28,32] provided information to calculate the overall effect size at the end of follow up, with totally 1,094 patients. Of these, only one was significant (MD = -0.48, 95% CI = -0.91--0.05) [19]. The analysis of the pooled data from the five trials demonstrated a reduction in HbA1c values at the end of follow up in favor of collaborative care (MD = -0.13, 95% CI = -0.46-0.19; *P =* 0.08 for heterogeneity; I^2^ = 51%) although this was not statistically significant (Figure 4).

### Diabetes clinical outcomes (HbA1c values) at 6 and 12 months follow up

**Figure 5:**
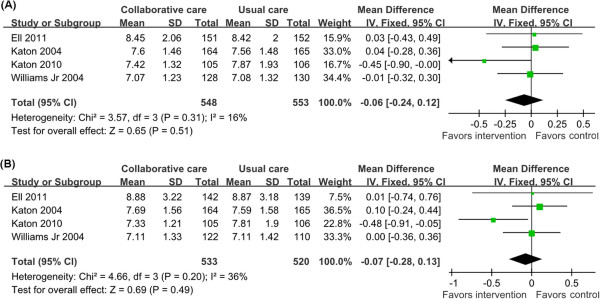
**Diabetes clinical outcomes (HbA1c values) at 6 and 12 months.** HbA1c values were estimated from meta-analysis of diabetic depression patients with collaborative care (intervention group) versus usual care (control group). **(A)** HbA1c values at 6 months. **(B)** HbA1c values at 12 months.

Four trials evaluated HbA1c values at 6 months follow up, with 1,101 patients to calculate the overall effect size [19,23,28,32]. There was a non-significant reduction in HbA1c values in favor of collaborative care (MD = -0.06, 95% CI = -0.24-0.12; *P =* 0.31 for heterogeneity; I^2^ = 16%) (Figure 5A).

Four trials evaluated HbA1c values at 12 months follow up, with 1,053 patients to calculate the overall effect size [19,23,28,32]. The analysis of the pooled data from the four trials demonstrated a reduction in HbA1c values at 12 months follow up in favor of collaborative care (MD = -0.07, 95% CI = -0.28-0.13; *P =* 0.20 for heterogeneity; I^2^ = 36%) although this was not statistically significant (Figure 5B).

### Adherence to antidepressant medication and oral hypoglycemic agent

**Figure 6:**
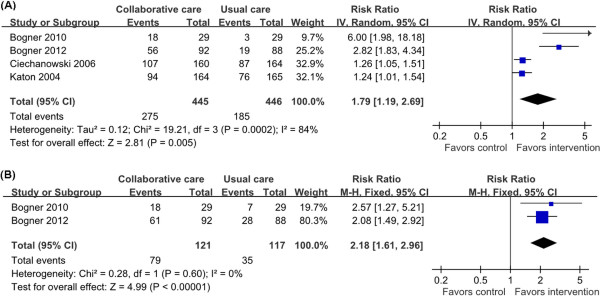
**Adherence to antidepressant medication and oral hypoglycemic agent.** Adherence to antidepressant medication and oral hypoglycemic agent were estimated from meta-analysis of diabetic depression patients with collaborative care (intervention group) versus usual care (control group). **(A)** Number of patients with adherence to antidepressant medication. **(B)** Number of patients with adherence to oral hypoglycemic agent.

Four trials reported rates of adherence to antidepressant medication, all provided information to calculate the overall effect size [23,26,27,31]. There were a total of 891 patients. All the four trials reported a significant improvement on adherence to antidepressant medication. The meta-analysis also indicated a statistical significant positive effect on rates of adherence to antidepressant medication (RR = 1.79, 95% CI = 1.19-2.69; *P <* 0.001 for heterogeneity; I^2^ = 84%) (Figure 6A).

Two trials provided information to calculate the pooled effect size on adherence to oral hypoglycemic agent, with a total of 238 patients [26,27]. Both of them showed a significant improvement on adherence to oral hypoglycemic agent. The pooled data indicated that collaborative care was associated with a significant improvement of adherence to oral hypoglycemic agent (RR = 2.18, 95% CI = 1.61-2.96; P = 0.60 for heterogeneity; I2 = 0%) (Figure 6B).

## Discussion

The present study comprehensively summarized current evidences from 8 RCTs to examine if collaborative care can work as a truly integrated intervention to improve both depression and diabetes outcomes, comparing with usual care. We found that collaborative care significantly improved depression outcomes, as well as adherence to antidepressant medication and oral hypoglycemic agent. Though there were significant heterogeneity in the meta-analyses, we found results from large RCTs were consistent [19,31,32], which strengthened the robustness of our conclusion.

Our study showed that collaborative care improved depression treatment response of diabetic patients with depression. All combined results showed positive effects on depression treatment response at 6 months, 12 months and the end of follow up. Moreover, the improvement in the outcome at the end of follow up compared favorably with response rates in collaborative care trials, which included patients with and without long term conditions. For example, a previous meta-analysis of 79 RCTs of collaborative care for depression showed a RR of 1.29 (95% CI = 1.18-1.41) [12] versus a RR of 1.33 (95% CI = 1.05-1.68) in our study.

Collaborative care model was also significantly associated with higher rates of both adherence to antidepressant medication (RR = 1.79, 95% CI = 1.19-2.69) and oral hypoglycemic agent (RR = 2.18, 95% CI = 1.61-2.96) in depressed patients with diabetes. These results are encouraging. Previous studies showed that depression was significantly associated with poor adherence to medication in diabetic patients [8,15,39]. In view of the challenges that faced by physicians in primary care practices, collaborative care are most likely helpful to improving adherence rate of medication among depressed patients with diabetes.

The effects of collaborative care on depression remission rates were limited at the end of follow up. According to our combined results, it seems that collaborative care did not benefit long term depression remission (RR = 1.15, 95% CI = 0.87-1.52). This data was mainly based on the crude RRs or crude odds ratios (ORs) that reported in original RCTs. Most of the authors of these original RCTs only reported the crude RRs or ORs without adjusting the potential confounders (for example, age, base line SCL-20 and HbA1c values). In study of Ell et al [32], the authors anticipated these confounders and provided the adjusted analysis for collaborative care versus usual care, which showed a signification increase in depression remission at the end of follow up (adjusted RR = 1.53, 95% CI =1.11-2.12). However, the data could not be combined since there was only one study reported the adjusted results of this outcome. In the future, the trialists are recommended to report both adjusted and unadjusted results to prevent the potential selection bias [40,41]. In addition, our meta-analysis showed that collaborative care also improved treatment remission rates at 6 months follow up, but this effect was modest.

Out meta-analyses showed that the improvements of outcomes of depression were not accompanied by significant differences in HbA1c values between patients in collaborative care and usual care group. HbA1c values were not affected at 6 months, 12 months and the end of follow up. Of the trials included in qualitative synthesis, three of them [19,26,27] had found that collaborative care was associated with improved HbA1c values, and two of them was significant [19,26]. Given that diabetic patients with depression usually have more macrovascular and microvascular complications and higher numbers of risk factors than diabetic patients without depression, the collaborative care that focuses on improving management of both depression and diabetes are likely to be needed in the future, to improve clinical outcomes at a population level in both of chronic illnesses [19].

### Limitations of the review

Combining the results from different trials generated significant variations in outcomes. In meta-analyses of primary outcomes, there existed a significant heterogeneity in the overall results of depression treatment response. The values of I^2^ are 54%, 52% and 59% for treatment response at 6 months, 12 months and the end of follow up respectively, which were all represented moderate levels of heterogeneity [42]. We therefore used random effects models to combine the data of these outcomes. Collaborative care is a kind of complex intervention with a considerable variability involving separate mechanisms, which is difficult to specify and define [29]. Nevertheless, compared with previous reviews [22,43], which using broader inclusion criteria for collaborative care, this review was based on more precise definition of collaborative care [12,30].

We were unable to collect addition information from authors who were responded for the original studies. There might be some missing data. In addition, using the *Cochrane Collaboration’s tool for assessing risk of bias*, we have identified methodological limitations in the studies. For example, all studies were rated as high risk of bias for blinding of participants and personnel.

Last but not least, this meta-analysis was based on 8 RCTs with 2,238 patients from different primary care practices in the United States, which might not be representative of all patients with both diabetes and depression around the world. Further research is needed to help clarify whether collaborative care can be implemented outside the United States.

## Conclusions

In summary, collaborative care model significantly improves depression outcomes and adherence to medication in diabetic patients with depression, comparing with usual care. Collaborative care is recommended for patients with both depression and diabetes in the future.

## Competing interests

The authors declare that they have no competing interests.

## Authors’ contributions

AG and YH conceived and designed the experiments. YH, AG, XW, TW and RC involved in the interpretation of the results. YH and AG performed the experiments. YH, XW, TW and RC analyzed the data. YH and AG wrote the paper. All authors read and approved the final manuscript.

## Pre-publication history

The pre-publication history for this paper can be accessed here:

http://www.biomedcentral.com/1471-244X/13/260/prepub

## Supplementary Material

Additional file 1 Search strategies. Click here for file

Additional file 2 Additional information of the included studies. Click here for file
