# Supplementary material for: Collaborative care for patients with depression and diabetes mellitus: a systematic review and meta-analysis
Source: BMC Psychiatry. 2013 Oct 14;13:260. doi: 10.1186/1471-244X-13-260 (PMC3854683; doi:10.1186/1471-244X-13-260)
Supplement: Additional file 1 — Search strategies. [file 1471-244X-13-260-S1.doc]

**Additional file 1 Search strategies**

***MEDLINE***

1. exp Depression/
2. exp Diabetes Mellitus/
3. diabet$.tw,ot.
4. (IDDM or NIDDM or MODY or T1DM or T2DM or T1D or T2D).tw,ot.
5. (non insulin$ depend$ or noninsulin$ depend$ or non insulin?depend$ or noninsulin?depend$).tw,ot.
6. (insulin$ depend$ or insulin?depend$).tw,ot.
7. or/2-7
8. disease management/
9. Disease management.mp.
10. exp Managed Care Programs/
11. managed care.mp.
12. (insurance and "case management").mp.
13. exp Patient Care Planning/
14. "patient care plan$".mp.
15. "Patient Centered Care$".mp.
16. "Multidisciplinary Care$".mp.
17. "nursing care plan$".mp.
18. "goals of care".mp.
19. "care goal".mp.
20. exp "Delivery of Health Care, Integrated"/
21. (integrated and (health$ or care$ or delivery or system$)).mp.
22. disease state management.mp.
23. Comprehensive Health Care/
24. "comprehensive health care".mp.
25. ((interdisciplin$ or multidisciplin$) and (care or health$ or delivery or system$)).mp.
26. Primary Nursing/
27. "primary nursing".mp.
28. "community based".mp.
29. Patient-Centered Care/
30. Patient Care Management/
31. (patient adj3 (care or management)).mp.
32. practice guideline/
33. education, medical, continuing/ or education, nursing, continuing/
34. exp community health services/
35. Primary Health Care/
36. "patient care team".mp.
37. "critical pathways".mp.
38. "case management".mp.
39. Collaboration/
40. Self Care/
41. (continuity adj3 "patient care").mp.
42. guideline$.mp.
43. "clinical protocol".mp.
44. "patient education".mp.
45. (self-care or "self care").mp.
46. reminder systems.mp. or Reminder Systems/
47. Health Education/
48. Health Promotion/
49. (health adj3 (education or promotion)).mp.
50. Community Health Planning/
51. ambulatory care.mp.
52. feedback.mp.
53. or/8-53
54. (clinical trial or controlled clinical trial or randomized controlled trial).pt.
55. (randomized or randomised).ab,ti.
56. placebo.ab,ti.
57. dt.fs.
58. randomly.ab,ti.
59. trial.ab,ti.
60. groups.ab,ti.
61. or/54-60
62. Animals/
63. Humans/
64. 62 not (62 and 63)
65. 61 not 64
66. 1 and 7 and 53 and 65

***Embase***

1. exp Depression/
2. exp Diabetes Mellitus/
3. diabet$.tw,ot.
4. (non insulin* depend* or noninsulin* depend* or non insulin?depend* or noninsulin?depend*).tw,ot.
5. (insulin* depend* or insulin?depend*).tw,ot.
6. (IDDM or NIDDM or MODY or T1DM or T2DM or T1d or T2D).tw,ot.
7. or/2-7
8. disease management/
9. Disease management.mp.
10. managed care/
11. managed care.mp.
12. (insurance and "case management").mp.
13. patient care planning/
14. "patient care plan$".mp.
15. "nursing care plan$".mp.
16. "goals of care".mp.
17. "care goal".mp.
18. integrated health care system/
19. (integrated adj5 (health$ or care$ or delivery or system$)).mp.
20. disease state management.mp.
21. health care/
22. "comprehensive health care".mp.
23. ((interdisciplin$ or multidisciplin$) adj5 (care or health$ or delivery or system$)).mp.
24. primary nursing/
25. "primary nursing".mp.
26. "community based".mp.
27. patient care/
28. (patient adj3 (care or management)).mp.
29. practice guideline/
30. medical education/
31. exp community care/
32. primary health care/
33. "patient care team".mp.
34. "critical pathways".mp.
35. "case management".mp.
36. self care/
37. Collaboration/
38. (continuity adj3 "patient care").mp.
39. guideline$.mp.
40. "clinical protocol".mp.
41. "patient education".mp.
42. (self-care or "self care").mp.
43. reminder system/
44. reminder systems.mp.
45. health education/
46. health promotion/
47. (health adj3 (education or promotion)).mp.
48. health care planning/
49. ambulatory care.mp.
50. feedback.mp.
51. or/8-50
52. Randomized Controlled Trial/
53. randomization/
54. Controlled Study/
55. Clinical Trial/
56. controlled clinical trial/
57. Double Blind Procedure/
58. Single Blind Procedure/
59. Crossover Procedure/
60. or/52-59
61. (clinica$ adj3 trial$).mp.
62. ((singl$ or doubl$ or trebl$ or tripl$) adj3 (mask$ or blind$ or method$)).mp.
63. exp Placebo/
64. placebo$.mp.
65. random$.mp.
66. ((control$ or prospectiv$) adj3 (trial$ or method$ or stud$)).mp.
67. (crossover$ or cross-over$).mp.
68. or/61-67
69. 60 or 68
70. exp ANIMAL/
71. Nonhuman/
72. Human/
73. 70 or 71
74. 73 not 72
75. 69 not 74
76. 1 and 7 and 51 and 75

***The Cochrane Library***

#1 MeSH descriptor Diabetes mellitus explode all trees

#2 diabet* in All Text

#3 (IDDM in All Text or NIDDM in All Text or MODY in All Text)

#4 (late in All Text and (onset in All Text near/6 diabet* in All Text))

#5 (maturity in All Text and (onset in All Text near/6 diabet* in All Text))

#6 (syndrom in All Text and (X in All Text near/6 diabet* in All Text))

#7 (hyperinsulin* in All Text or (insulin in All Text and sensitiv* in All Text))

#8 (insulin* in All Text and secret in All Text and dysfunction* in All Text)

#9 (impaired in All Text and glucose in All Text and toleran* in All Text)

#10 (glucose in All Text and intoleran* in All Text)

#11 MeSH descriptor Glucose Intolerance explode all trees

#12 (insulin* in All Text and resist* in All Text)

#13 ((non in All Text and insulin* in All Text and depend* in All Text) or (noninsulin* in All Text and depend* in All Text) or (non in All Text and insulin?depend* in All Text) or noninsulin?depend* in All Text)

#14 MeSH descriptor Insulin resistance explode all trees

#15 ((insulin* in All Text and depend* in All Text) or insulin?depend* in All Text)

#16 or/1-15

#17 “Disease management”

#18 MeSH descriptor Managed Care Programs explode all trees

#19 “managed care”

#20 insurance and “case management”

#21 MeSH descriptor Patient Care Planning explode all trees

#22 “patient care plan*”

#23 “nursing care plan*”

#24 “goals of care”

#25 “care goal”

#26 (integrated and (health* or care* or delivery or system*))

#27 “disease state management”

#28 “comprehensive health care”

#29 ((interdisciplin* or multidisciplin*) and (care or health* or delivery or system*))

#30 “primary nursing”

#31 “community based”

#32 MeSH descriptor Patient-Centered Care explode all trees

#33 “patient care”

#34 “patient management”

#35 MeSH descriptor Community Health Services explode all trees

#36 “patient care team”

#37 “critical pathways”

#38 “case management”

#39 continuity NEAR/3 “patient care”

#40 guideline*

#41 “clinical protocol”

#42 “patient education”

#43 self-care or “self care”

#44 MeSH descriptor Reminder Systems explode all trees

#45 “reminder system*”

#46 MeSH descriptor Health Promotion explode all trees

#47 health NEAR/3 (educat* or promot*)

#48 “ambulatory care”

#49 feedback

#50 or/17-49

#51 16 and 50

#52 MeSH descriptor Diabetes mellitus explode all trees

#53 51 and 52

***PsycINFO***

1. exp Depression/
2. exp Diabetes Mellitus/
3. diabet$.tw,ot.
4. (non insulin* depend* or noninsulin* depend* or non insulin?depend* or noninsulin?depend*).tw,ot.
5. (insulin* depend* or insulin?depend*).tw,ot.
6. (IDDM or NIDDM or MODY or T1DM or T2DM or T1d or T2D).tw,ot.
7. or/2-7
8. disease management/
9. Disease management.mp.
10. managed care/
11. managed care.mp.
12. (insurance and "case management").mp.
13. patient care planning/
14. "patient care plan$".mp.
15. "nursing care plan$".mp.
16. "goals of care".mp.
17. "care goal".mp.
18. integrated health care system/
19. (integrated adj5 (health$ or care$ or delivery or system$)).mp.
20. disease state management.mp.
21. health care/
22. "comprehensive health care".mp.
23. ((interdisciplin$ or multidisciplin$) adj5 (care or health$ or delivery or system$)).mp.
24. primary nursing/
25. "primary nursing".mp.
26. "community based".mp.
27. patient care/
28. (patient adj3 (care or management)).mp.
29. practice guideline/
30. medical education/
31. exp community care/
32. primary health care/
33. "patient care team".mp.
34. "critical pathways".mp.
35. "case management".mp.
36. self care/
37. Collaboration/
38. (continuity adj3 "patient care").mp.
39. guideline$.mp.
40. "clinical protocol".mp.
41. "patient education".mp.
42. (self-care or "self care").mp.
43. reminder system/
44. reminder systems.mp.
45. health education/
46. health promotion/
47. (health adj3 (education or promotion)).mp.
48. health care planning/
49. ambulatory care.mp.
50. feedback.mp.
51. or/8-50
52. Randomized Controlled Trial/
53. randomization/
54. Controlled Study/
55. Clinical Trial/
56. controlled clinical trial/
57. Double Blind Procedure/
58. Single Blind Procedure/
59. Crossover Procedure/
60. or/52-59
61. (clinica$ adj3 trial$).mp.
62. ((singl$ or doubl$ or trebl$ or tripl$) adj3 (mask$ or blind$ or method$)).mp.
63. exp Placebo/
64. placebo$.mp.
65. random$.mp.
66. ((control$ or prospectiv$) adj3 (trial$ or method$ or stud$)).mp.
67. (crossover$ or cross-over$).mp.
68. or/61-67
69. 60 or 68
70. exp ANIMAL/
71. Nonhuman/
72. Human/
73. 70 or 71
74. 73 not 72
75. 69 not 74
76. 1 and 7 and 51 and 75
