# Supplementary material for: Collaborative care for patients with depression and diabetes mellitus: a systematic review and meta-analysis
Source: BMC Psychiatry. 2013 Oct 14;13:260. doi: 10.1186/1471-244X-13-260 (PMC3854683; doi:10.1186/1471-244X-13-260)
Supplement: Additional file 2 — Additional information of the included studies. [file 1471-244X-13-260-S2.doc]

**Additional file 2 Additional information of the included s**tudies.

| **Author** | **Year** | **Eligible patients** | **Description of “Collaborative care”** | **Description of “control”** | **Main outcomes included** |
| --- | --- | --- | --- | --- | --- |
| Bogner et al. [26] | 2012 | 1. Aged 30 years and older. 2. Diagnosis of depression: A current prescription for an antidepressant. 3. Diagnoses of diabetes: A current prescription for an oral hypoglycemic agent. | Integrated Management   1. A multi-professional patients care (PCP, nurse, psychologist) 2. A structured management plan (GBTR, IA, TPA) 3. Scheduled patient follow up (IPC, telephone) 4. Enhanced inter-professional communication | A usual-care that patients received care for depression and diabetes from their PCP. | 1. HbA1c values (6, 12 wk) 2. Adherence to antidepressant medication (6, 12 wk) 3. Adherence to oral hypoglycemic agent (6, 12 wk) |
| Bogner et al. [27] | 2010 | 1. Aged 50 and older, African Americans. 2. Diagnosis of depression: A diagnosis of depression by primary care physician or a prescription for an antidepressant within the past year. 3. Diagnosis of diabetes: An HbA1c >7 at last primary care office visit or a prescription for an oral hypoglycemic agent with the past year. | Integrated Management   1. A multi-professional patients care (PCP, nurse, psychologist) 2. A structured management plan (GBTR, IA, TPA) 3. Scheduled patient follow up (IPC, telephone) 4. Enhanced inter-professional communication | A usual-care that patients received care for depression and diabetes from their PCP. | 1. HbA1c values (6 wk) 2. Adherence to antidepressant medication (6 wk) 3. Adherence to oral hypoglycemic agent (6 wk) |
| Ciechanowski et al. [36] | 2006 | 1. Aged 18 and older. 2. Diagnosis of depression: Scored >=10 on the PHQ-9 and persistent symptoms evidenced by SCL-20>1.1. 3. Diagnosis of diabetes: Diagnosis made by primary care physicians and a current prescription for an oral hypoglycemic agent. | Program to Encourage Active, Rewarding Lives for Seniors (PEARLS)   1. A multi-professional patients care (PCP, nurse, psychologist) 2. A structured management plan (ISCG, TPA) 3. Scheduled patient follow up (in-home sessions, telephone) 4. Enhanced inter-professional communication | An enhanced usual-care that patients received care for depression and diabetes from their PCP. | 1. Adherence to antidepressant medication (12 mo) 2. Adherence to oral hypoglycemic agent (12 mo) |
| Ell et al. [38] | 2011 | 1. Aged 18 and older. 2. Diagnosis of depression: Endorsed one of the 2 cardinal depression symptoms more than half the days to nearly every day over the last two weeks and scored >=10 on the PHQ-9 indicating clinically significant depression. 3. Diagnosis of diabetes: Diagnosis made by primary care physicians. | Multifaceted Diabetes and Depression Program (MDDP)   1. A multi-professional patients care (PCP, nurse, psychologist) 2. A structured management plan (IA, ISCG, TPA) 3. Scheduled patient follow up (PSG, telephone) 4. Enhanced inter-professional communication | An enhanced usual-care that patients received care for depression and diabetes from their PCP. | 1. HbA1c values (6, 12, 24 mo) 2. Treatment Response (>=50% decrease in SCL-20 score) (6, 12, 24 mo) 3. Complete Remission (SCL-20 score <0.5) (6, 12, 24 mo) |
| Katon et al. [19] | 2010 | 1. Diagnosis of depression: Patients with PHQ-2 scores of 3 or more or higher on PHQ-9. 2. Diagnosis of diabetes: ICD-9 codes for diabetes. | Collaborative care   1. A multi-professional patients care (PCP, nurse, psychologist) 2. A structured management plan (PST, TPA) 3. Scheduled patient follow up (IPC, telephone) 4. Enhanced inter-professional communication | An enhanced usual-care that patients received care for depression and diabetes from their PCP. | 1. Treatment Response (>=50% decrease in SCL-20 score) (6, 12 mo) 2. HbA1c values (6, 12 mo) |
| Katon et al. [23] | 2004 | 1. Diagnosis of depression: Patients with PHQ-9 scores of 10 or higher. 2. Diagnosis of diabetes: ICD-9 codes for diabetes. | Stepped collaborative care   1. A multi-professional patients care (PCP, nurse, psychologist) 2. A structured management plan (PST, SCEM, ISCG, TPA) 3. Scheduled patient follow up (IPC, telephone) 4. Enhanced inter-professional communication | A usual-care that patients received care for depression and diabetes from their PCP. | 1. Treatment Response (>=50% decrease in SCL-20 score) (6, 12 mo) 2. HbA1c values (6, 12 mo) 3. Adherence to antidepressant medication (12 mo) |
| Kinder et al. [37] | 2006 | 1. Diagnosis of depression: Scored >=10 on the PHQ-9 and persistent symptoms evidenced by SCL-20>1.1. 2. Diagnosis of diabetes: ICD-9 codes for diabetes. | An individualized stepped-care depression treatment program   1. A multi-professional patients care (PCP, nurse, psychologist) 2. A structured management plan (PST, TPA) 3. Scheduled patient follow up (IPC, telephone) 4. Enhanced inter-professional communication | A usual-care that patients received care for depression and diabetes from their PCP. | 1. Treatment Response (>=50% decrease in SCL-20 score) (6, 12 mo) 2. Complete Remission (SCL-20 score <0.5) (6, 12 mo) |
| Williams Jr et al. [28] | 2004 | 1. Aged 60 years and older. 2. Diagnosis of depression: Structured psychiatric interview and DSM. 3. Diagnosis of diabetes: Diagnosis made by primary care physicians by self-report of patients. | Improving Mood Promoting Access to Collaborative Treatment (IMPACT)   1. A multi-professional patients care (PCP, nurse, psychologist) 2. A structured management plan (PST, SCEM, TPA) 3. Scheduled patient follow up (IPC, telephone) 4. Enhanced inter-professional communication | A usual-care that patients received care for depression and diabetes from their PCP. | 1. HbA1c values (6, 12 mo) 2. SCL-20 depression score (6, 12 mo) |

Abbreviations: CHD = coronary heart disease, DSM = Diagnostic and Statistical Manual, GBTR = guideline based treatment recommendations, HbA1c = Hemoglobin A1c, IA = individualized assessment, ICD-9 = International Classification of Diseases, 9^th^ Revision, IPC = in-person contact, ISCG = individualized self-care goals, mo = month, PCP = primary care providers, PHQ = patient health questionnaire, PSG = patient support group, PST = Problem-solving treatment, SCEM = Self-care educational materials, SCL-20 = Symptom Checklist-20, TPA = treatment plan adjustments, wk = week.
